# Telecoupled urban demand from West African cities causes social-ecological land use transformation in Saharan oases

**DOI:** 10.1371/journal.pone.0289694

**Published:** 2023-09-08

**Authors:** Kira Fastner, Salouhou Djibrilla, Thanh Thi Nguyen, Andreas Buerkert

**Affiliations:** 1 Organic Plant Production and Agroecosystems Research in the Tropic and Subtropics, Organic Agricultural Sciences, Universität Kassel, Witzenhausen, Germany; 2 École Bagzam, Emalawlé, Agadez, République du Niger; Kerala University of Fisheries and Ocean Studies, INDIA

## Abstract

Little is known about the long-distance telecoupling effects of urban food demands on land use changes (LUCs) in remote oases of the Southern Sahara. Using the example of two typical oasis settlements on Mont Bagzam in the southern Aϊr Mountains of Niger which are linked to regional and global markets by an unpaved road since 2015, this study aimed at analyzing time trajectories of LUCs and related changing agricultural production patterns. LUCs were quantified for 1955 to 2022 using GIS-based mapping of agriculture and natural vegetation based on historical aerial photographs, CORONA and multi-spectral satellite images, and high resolution drone-based surveys. The results show a major increment in actively used agricultural land in the 850 ha watershed of the two oases from 11 ha in 1955 to 13 ha in 2003 and 68 ha in 2022 as well as the addition of 92 irrigation wells to 16 existing ones between 2003 and 2022. LUCs and evapotranspiration calculated from climatic data of a local weather station allowed to estimate changes of irrigation water needs in the selected watershed. While annual precipitation averages only 214 mm, local reference evapotranspiration may reach 1,476 mm year^-1^. Therefore, the additional annual irrigation water needs for the newly established fields between 2003 and 2022 cultivated to cash crops rose by 696 million l. To detect LUC effects on soil quality, soil samples of onion and garlic fields of different ages were collected employing a false-time-series approach. Results reveal increasing soil pH and salt concentrations and falling ground water tables, which reflects a negative water balance and ground water extraction above recharge levels. Our study provides evidence that the newly established telecoupled production systems on Mont Bagzam threaten the sustainability of existing local agricultural production and related livelihoods of agro-pastoralists.

## Introduction

Oasis agriculture dates back to prehistoric times and is one of the most durable modes of farming and managing scarce natural resources [1–3], such as fertile land, labor, and water, the three core determinants of human survival until today [4]. Oases are usually defined as agricultural systems in (hyper-) arid environments created by human activities through harnessing water resources by channeling of surface flow, tapping of springs, or draining of water-soaked subsoil [1,5]. Once water is made accessible, leveled field gardens are irrigated through more or less intricate systems of channels from often distant water sources [5]. Recent evidence shows the heavy dependency of these social-ecological systems [6,7] on the preservation of integrated crop-livestock husbandry that has determined soil fertility, plant diversity, and water quality for centuries, if not millennia [8–12].

While the role of North African and Arabian oasis settlements as remote production locations for local food production and often ancient trading hubs is well documented, there is very little data on the evolution of oases in the Saharan Desert of Niger. This is all the more surprising as they have for long played a significant role for transnational trading of goods [2,13,14] and serve as showcases for human adaptability to harsh environments.

Located at the southern fringe of the Saharan Desert, the Aïr Mountains with their highest peak Idoukal-n-Taghès (2022 m above sea level (asl)) on the massif of Mont Bagzam, are sparsely populated by sedentary Kel Ewey Touareg settling in different oases and practicing agro-pastoralism since many centuries [15]. The spatial isolation of the area and harsh climatic conditions with average annual rainfall ranging from only 67–348 mm [16] restricted the population to practice sheep and goat herding combined with small-scale subsistence-oriented, irrigated agriculture, and caravan trade for salt, dates, and millet [16].

For many decades on Mont Bagzam traditional oasis gardens were either irrigated by spring water channeled through canals to the fields or by human- or animal-powered draw wells limited to fetching groundwater close to the surface and irrigating small basins. Cultivated subsistence cereals comprised wheat (*Triticum aestivum* L.), barley (*Hordeum vulgare* L.), millet (*Pennisetum glaucum* L.), and alfalfa (*Medicago sativa* L.). Traditionally also grown on the ancient, deep volcanic soils are vegetables such as potato (*Solanum tuberosum* L.), onion (*Allium cepa* L.), and cabbage (*Brassica oleracea* L.) sometimes intercropped with fruit trees such as citrus (*Citrus* L.), fig (*Ficus carica* L.), lime (*Citrus × latifolia)*, pomegranate (*Punica granatum* L.), and date palm (*Phoenix dactylifera* L.). The latter are part of the classical three-story agroforestry arrangement fostering agricultural productivity through decreasing air temperature and maintaining higher air humidity inside the oasis [17–19]. As typical for such systems, after crop harvest sheep and goats stubble-graze oasis fields and are herded on the sparse but vast surrounding pastures from where they funnel plant nutrients and carbon to the agricultural areas [8,20]. Today small ruminants still play an important role for sustaining livelihoods and productivity of these oasis, however, their manure is increasingly complemented by mineral fertilizers and applied to newly established cash-crop fields where application is more profitable for farmers.

**Fig 1:**
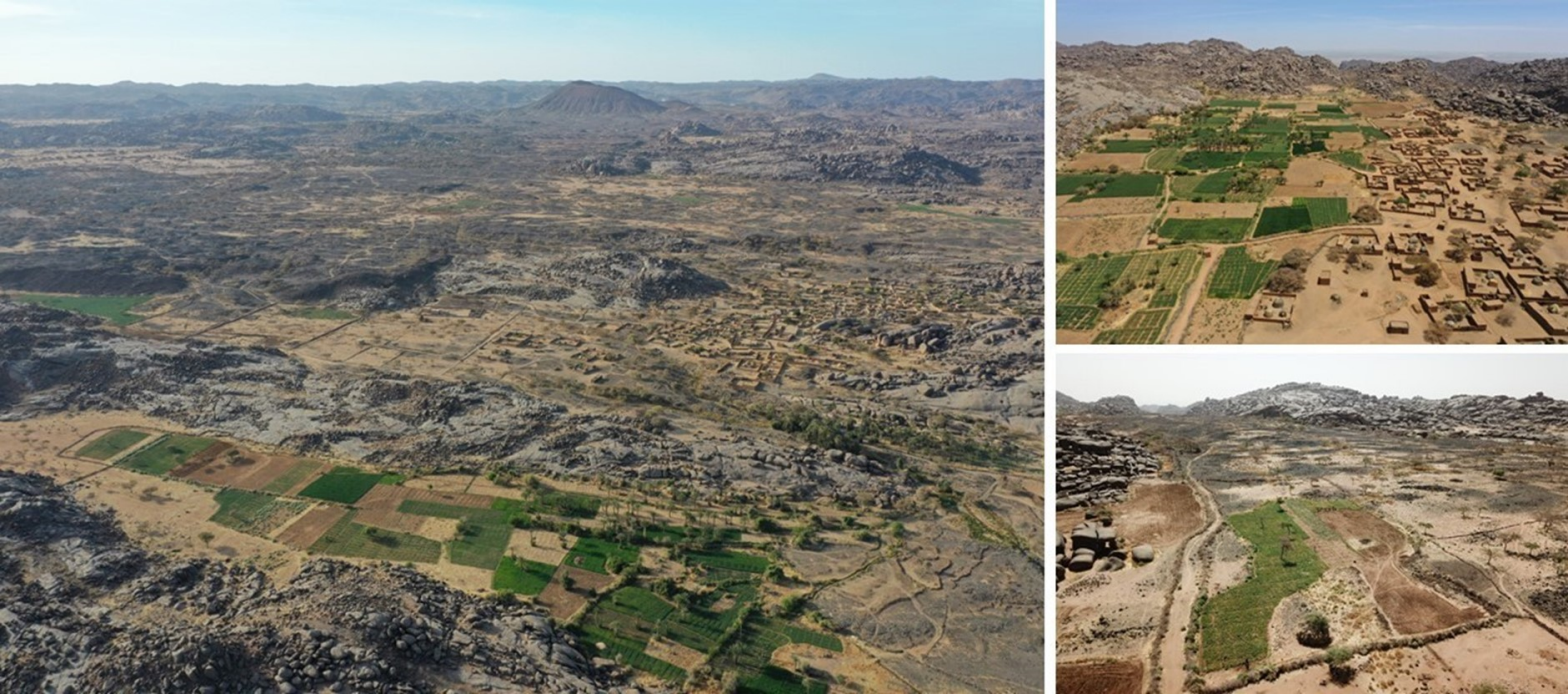
Aerial photographs of oases on Mont Bagzam, Niger. Oasis gardens of Emalawlé in the foreground with the village and newly established, market-oriented vegetable fields in the background (left), oasis gardens of Aoukadédé (upper right) and newly established fields in the watershed between Emalawlé and Aoukadédé (lower right) on Mont Bagzam in the Aïr Mountains of Niger.

With the establishment of long-distance road infrastructure for cars and trucks during the past decades, caravan trade has lost much of its place, while export of marketable crops to distant markets provides new sources of income. In 2015 market links were strongly enhanced by the locally initiated construction of a motorable road from Mont Bagzam’s central village of Bagzam n’Amass to the foothill village of Tokadi which facilitates trading to the nearby market hubs of Tabelot and Agadez. At the same time the arrival of mobile phone communication has connected formerly secluded communities on Mont Bagzam to the global information network, resulting in quickly changing perceptions of the environment and livelihoods, especially of the younger generation. It has also allowed local producers to follow the ups and downs of market prices for agricultural inputs and outputs. This has contributed to today’s managed mosaic in land use where marginal pasture lands are dissected by an increasing number of irrigated fields (Fig 1) cultivated mainly with the cash crops onion, garlic, and some potato.

The objective of our study was to analyze the far-reaching effects of the global market economy and growing urban centers in West Africa on the sustainability of land use in spatially isolated areas undergoing changing agricultural practices. To assess the magnitude of this regime shift from subsistence-oriented agro-pastoral ecosystems into increasingly market-oriented ones and its consequences for the livelihoods on Mont Bagzam’s rapidly growing population, we hypothesized that (i) a time trajectory of available remote sensing datsets allows to detect land use changes (LUCs) at sufficient resolution to match them with survey records and (ii) the observed changes in land use patterns affect the sustainability of the agro-ecosystems as indicated by increased pressure on water resources and soil quality.

## Materials and methods

### Agroecological setting

**Fig 2:**
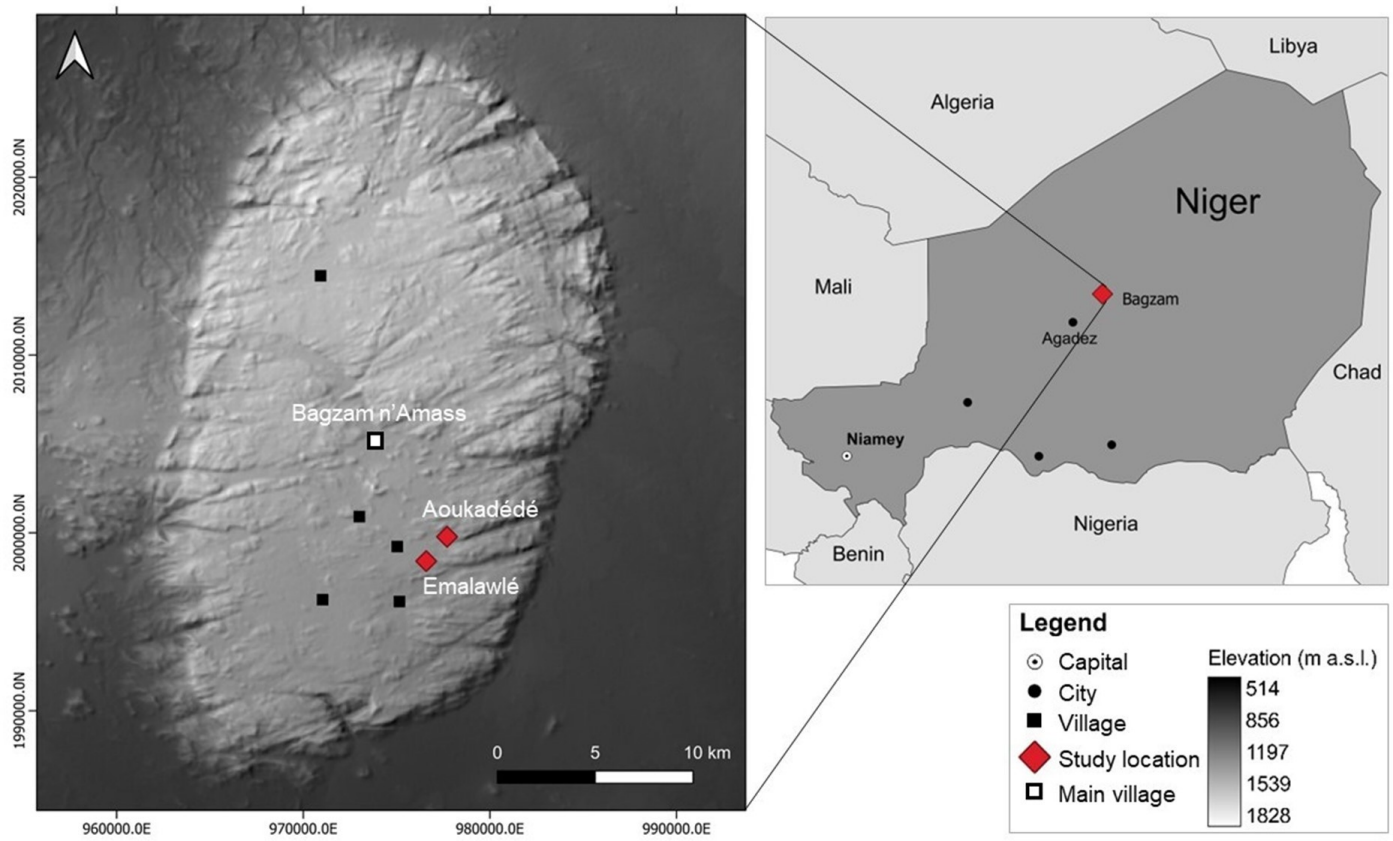
Study area. Location of the study sites on Mont Bagzam, Niger (Digital Elevation Model (DEM), 2012) [22].

Mont Bagzam in the south of the Aϊr Mountains of Niger consists of a dissected and heavily eroded plateau of peralkaline granites intersected by metamorphic rocks and ancient Cenozoic volcano chimneys surrounded by vast areas of lawa debris. The plateau comprises twelve small-scale oasis systems, which are partly connected by unpaved roads (Fig 2). Despite unknown emigration to the lowlands, population numbers on Mont Bagzam tripled from around 2,000 inhabitants in 1973 [15] to 6,000 in 2021. Bagzam n’Amass, the administrative center of Mont Bagzam comprises an area of 1,240 ha (17°43’31.28”N 8°45’9.73”E, 1,550 m asl).

Our study focused on the approx. 100-year-old oasis of Emalawlé (17°39’36.0”N 8°46’22.1”E; 1480 m asl) and the 70-year-old oasis of Aoukadédé (17°40’35.5”N 8°47’01.5”E, 1480 m asl) with their watersheds comprising a total area of 820 ha. The old oasis centers contain irrigated gardens divided over generations according to traditional maternal inheritance rights. This takes account of traditional Tuareg livelihood strategies in which husbands are absent during a sizeable part of the year practicing caravan trade, while their wives are responsible for the household and childcare. In Emalawlé agricultural plots are irrigated in 5–7 day intervals from one collectively managed spring, while fields in Aoukadédé and most newly established fields are daily irrigated from individually managed wells nowadays almost all equipped with motor pumps rather than the traditional animal driven, low-capacity systems (*sāqiyah*) of fetching ground water.

### Climatic conditions

**Fig 3:**
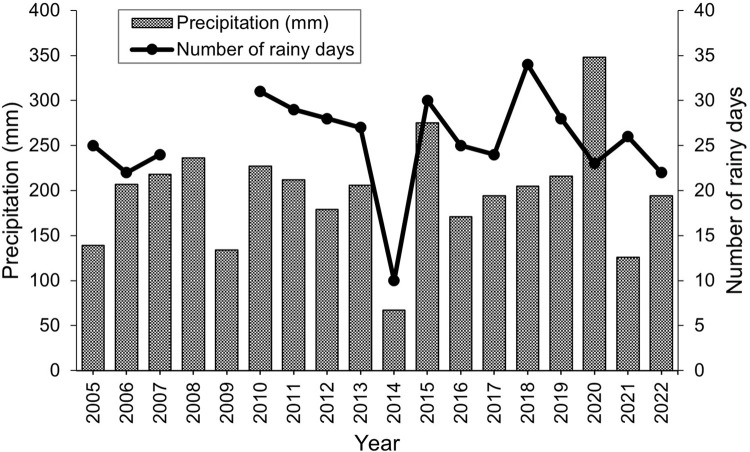
Annual precipitation. Annual precipitation (2005–2021) in Emalawlé, Mont Bagzam, Niger.

Solar radiation, sun air temperature, and shade air temperature/humidity data were recorded in 30 min intervals with a HOBO MX2202 data logger installed on top of a meteo box containing a HOBO U23-002 Pro v2 data logger (Onset, Bourne, MA, USA) installed in July 2021 in the village of Emalawlé. These data complemented rainfall records determined with a traditional pluviometer from 2005–2022 (Fig 3).

**Fig 4:**
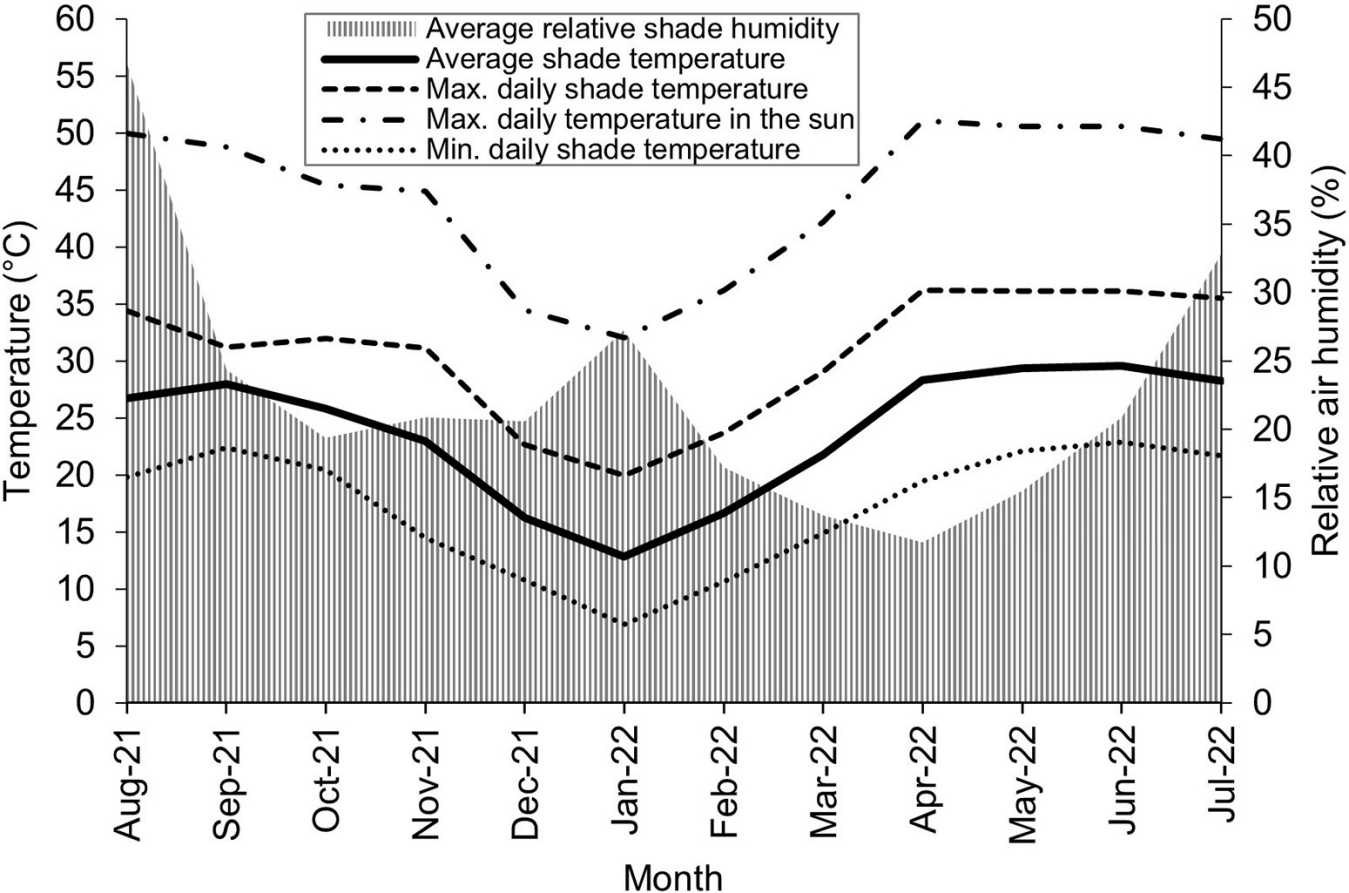
Temperature and air humidity. Monthly air temperatures and moisture (August 2021 –August 2022) in Emalawlé, Mont Bagzam, Niger.

Average monthly temperatures varied between 13°C and 30°C whereby summer temperatures may reach 40°C in the shade and 57°C in full sunshine while winter temperatures can fall down to -2°C. The average daily solar radiation amounts to 51,700 LUX between 6:30 am and 6:30 pm, whereby maximum radiation values may reach 130,000 LUX. Relative air humidity is highest during the short rainy season in July/August with a daily maximum of 76% in August and in January with 40%, while during the other months of the year it remains < 25% (Fig 4).

### Evapotranspiration

For the assessment of changes in crop water requirements due to the establishment of new large-scale fields in the studied watershed, the monthly crop evapotranspiration demand under optimal agronomic and soil water conditions was estimated. Most of these new fields are cultivated with a simple cash crop rotation of garlic (*Allium sativum*, November–May) and onion (July–October) followed by a short fallow period in June. Parts of some fields are also cultivated with potato from February until November. To simplify the assessment of the crop evapotranspiration demand of these fields, we assumed that they comprised a crop rotation of irrigated garlic and onion without fallow periods.

Therefore, the potential crop evapotranspiration (ET_c_) for onion and garlic was computed in monthly time intervals as:

$${ET}_{c}=k_{c}{ET}_{0}$$ (1)

where ET_0_ is the reference evapotranspiration (mm day^-1^) and k_c_ is the crop coefficient that depends on crop type and development stage. ET_0_ refers to the evapotranspiration from a well-watered hypothetical grass surface with a fixed crop height, albedo and surface resistance [21].

The crop coefficients k_c_ for four growth stages (initial stage, crop development, mid-season, and late season) were defined using the single crop coefficient approach [21] and adjusted to match the observed management and climate conditions. The length of the four crop development stages, equal for both crops, was chosen based on survey results and site-specific field observations (Table 1). Initially, crop coefficients were kept constant at the level of k_c ini_ whereby during crop development crop coefficients increased by constant daily rates to the level given by k_c mid_. For the mid-season period, crop coefficients were constant at k_c mid_ and for the late season stage, crop coefficients were assumed to decline steadily to the value given by k_c end_ (Table 1).

**Table 1:** Growing periods and crop coefficients of garlic and onion. Length of initial growing period (L_ini_), crop development period (L_dev_), mid-season period (L_mid_), late season period (L_late_), and total length of growing period (L_tot_) in days, crop coefficients for the initial period (k_c ini_), mid season (k_c mid_), end season (k_c end_), and average crop coefficient over the growing season (k_c avg_) for onion and garlic on Mont Bagzam, Niger.

Crop	Length of growing periods (day)					Crop coefficients			
	L_ini_	L_dev_	L_mid_	L_late_	L_tot_	k_c ini_	k_c mid_	k_c end_	k_c avg_
Garlic	20	30	90	40	180	0.70	1.00	0.70	0.88
Onion	20	30	90	40	180	0.70	1.05	0.20	0.89

For the calculation of reference evapotranspiration (ET_0_, mm month^-1^) monthly averages of the previously described climatic measurements of shade air temperature (daily minimum, maximum and average), solar radiation, shade humidity, and wind speed were used (Table 2).

**Table 2:** Overview of climatic data. Monthly averages of daily maximum temperature (T_max_), daily minimum temperature (T_min_), daily average temperature (T_avg_), daily solar radiation (R_s_), daily maximum relative humidity (RH_max_), daily minimum relative humidity (RH_min_), and monthly sum of effective precipitation (P_e_) at Emalawlé, Mont Bagzam, Niger.

Month	T_max_ (°C)	T_min_ (°C)	T_avg_ (°C)	R_s_ (MJ	RH_max_ (%)	RH_min_ (%)	P_e_ (mm)
				m^-2^ day^-1^)			
Aug-21	34.4	19.8	26.7	16.3	76	23	32
Sep-21	31.2	22.3	27.1	17.8	35	20	0
Oct-21	31.9	20.5	25.8	16.2	28	13	0
Nov-21	31.1	14.5	23.0	14.5	33	12	0
Dec-21	22.7	10.7	16.2	13.5	31	13	0
Jan-22	19.9	6.9	12.8	14.4	40	17	0
Feb-22	23.7	10.6	16.6	16.3	26	10	0
Mar-22	29.0	14.9	21.8	16.9	21	8	0
Apr-22	36.2	19.5	28.3	18.4	20	7	0
May-22	36.1	22.1	29.3	17.7	25	10	0
Jun-22	36.1	22.9	29.6	16.6	36	12	2
Jul-22	35.5	21.7	28.2	15.8	57	17	11
Annual average*	30.7	17.2	23.8	16.2	36	13	45

* Denotes the annual sum of precipitation in 12 months.

ET_0_ from August 2021 –August 2022 was computed with the FAO Penman-Monteith equation [21]:

$${ET}_{0}=\frac{0.408\Delta (R_{n}-G)+\gamma \frac{900}{T+273}u(e_{s}-e_{a})}{\Delta +\gamma (1+0.34u)}$$ (2)

where R_n_ stands for the net radiation at the crop surface (MJ m^-2^ day^-1^), G for the soil heat flux density (MJ m^-2^ day^-1^), T is the mean daily air temperature (°C), u the wind speed at 2 m height (m s^-1^), e_s_ the saturation vapor pressure (kPa), e_a_ the actual vapor pressure (kPa), ∆ the slope of the vapor pressure curve (kPa°C^-1^), and γ stands for a psychrometric constant (kPa°C^-1^). Average wind speed (u) was set to 2 m s^-1^ and soil heat flux (G) to 0 as its magnitude is usually small [21]. Values of the slope of the vapor pressure curve (∆) for different mean air temperatures (T) and the psychrometric constant (γ) of 0.056 for an altitude of 1,500 m were also used [21].

Net radiation was computed as the difference between incoming net short wave radiation (R_ns_) and the outgoing net long wave radiation (R_nl_):

$$R_{n}=R_{ns}-R_{nl}$$ (3)

Net short wave radiation was calculated as:

$$R_{ns}=(1-\alpha )R_{s}$$ (4)

where α is a reflection coefficient set to 0.23 and R_s_ stands for the incoming short wave radiation (MJ m^-2^ day^-1^). Net outgoing long wave radiation (R_nl_) was determined as:

$$R_{nl}=\sigma \left(\frac{T_{max,K}^{4}+T_{min,K}^{4}}{2}\right)(0.34-0.14\sqrt{e_{a}})\left(1.35\frac{R_{s}}{R_{s0}}-0.35\right)$$ (5)

where σ stands for the Stefan–Boltzmann constant with 4.903 x 10^−9^ MJ K^-4^ m^-2^ day^-1^, T_max,K_ for the daily maximum temperature (K), and T_min,K_ for the daily minimum temperature (K). The short wave radiation R_s0_ on a clear-sky day (MJ m^-2^ day^-1^) was set equal to R_s_ since on Mount Bagzam clouds usually only occur during the short rainy season.

As the mean saturation vapor pressure (e_s_) is closely related to air temperature, it was calculated as:

$$e_{s}=\frac{e{}^{\circ}(T_{max})+e{}^{\circ}(T_{min})}{2}$$ (6)

with the saturation vapor pressure at the mean daily maximum (e°(T_max_)) and minimum (e°(T_min_)) air temperatures. The values of e°(T_max,min_) were also taken from a reference table [21].

The actual vapor pressure (e_a_) can be calculated from the relative humidity:

$$e_{a}=\frac{e{}^{\circ}(T_{min})\frac{{RH}_{max}}{100}+e{}^{\circ}(T_{max})\frac{{RH}_{min}}{100}}{2}$$ (7)

where RH_max_ stands for the maximum relative humidity (%) and RH_min_ for the minimum relative humidity (%).

For verification of calculated ET a real “pan” evaporation (ET_pan_) was measured using a water bucket (height of 30 cm, diameter of 32 cm, filled with 21 l of water and covered with a light mosquito net to deter birds) since a standard Class-A evaporation pan was not available [21]. Monthly differences in water levels (solely from evaporation) over a timeframe of one year (November 2021–2022) were recorded. ET_pan_ is related to ET_0_ by an empirically derived pan coefficient (K_p_) and was therefore computed as:

$${ET}_{0\;pan}=K_{p}{ET}_{pan}$$ (8)

where the pan coefficient (K_p_) was defined as 0.45 for a low humidity area, with medium wind speed and a dry fallow area [21].

### Oasis mapping

#### Image pre-processing

Time series analyses of remote sensing data were performed using an aerial photograph from 1955, a CORONA image from 1967, a total of six multi-spectral Landsat scenes between 1987 and 2021, a Maxar Technologies image from 2003, CNES/Airbus images from 2016 and 2019, and drone images from 2022 taken with a DJI Mini 2. Multi-spectral Landsat scenes and panchromatic CORONA images were downloaded from USGS’s Earth Explorer [22], the Maxar Technologies image and CNES/Airbus images from Google Earth Pro [23]. The aerial photograph with a scale of 1/50,000 was purchased from Institut National de l’Information Géographique et Forestière (IGN, Paris, France) and a drone orthomosaic was generated with DroneLink [24] for the cultivated areas of the two villages (820 ha, Table 3).

**Table 3:** Image data sources. Sources of remote sensing data for the study area on Mont Bagzam in the Aïr Mountains of Niger.

Sensor/Image	Resolution	Date	Data Source
Aerial photograph	1.7 m	1955-10-23	IGN France
CORONA	2.7 m	1967-07-01	[22]
Landsat 5 TM+	30 m	1987-10-10 1994-10-08	[22]
Landsat 8 OLI	30 m	2014-09-02 2016-09-07 2018-09-13 2021-09-21	[22]
Maxar Technologies CNES/Airbus	1.7 m 1.7 m 1.2 m	2003-08-01 2016-10-24 2019-08-31	Google, Maxar Technologies [23] Google, CNES/Airbus [23]
Drone DJI Mavic Mini 2	0.5 m	2022-08-10	Personal data

For analysis of Landsat scenes, images of September/October were chosen since a preliminary comparison of datasets for the different seasons had shown vegetation cover to be highest after the typically heavy August rainfalls therefore allowing better classification and comparison across years. This made the image availability to be rather restricted as also cloud cover is typically highest during this time of the year. Landsat 7 images were excluded from the dataset to prevent possible reflectance differences to Landsat 8 images and thus biases in the vegetation index [25].

Image (pre)processing was performed using the open-source Quantum GIS software (QGIS vers. 3.10). Thereby Landsat scenes were atmospherically corrected with the Semi-Automatic Classification Plugin [26] which accounts for effects of atmospheric errors induced by water vapor and aerosols that interact with the electromagnetic radiation and may lead to scattering and absorption of light [27]. With their resolution of < 3 m the black and white aerial photograph and CORONA image of 1955 and 1967 as well as the true color drone images were georeferenced and subsequently used for detailed manual classification of the study sites.

#### Determination of temporal changes in vegetation

As an indicator for live green vegetation monitoring, the Normalized Difference Vegetation Index (NDVI) is commonly used since the respective red (R) and near infrared (NIR) satellite bands contain over 90% of the vegetation information [28]:

NDVI=(NIR−R)/(NIR+R) (9)

By using Eq (9) NDVI values were computed for the Landsat scenes of the selected area on Mont Bagzam and compared over a time span of 44 years. Thereby it is understood that time series of NDVI values can indicate changes in vegetation due to variation in rainfall, human activities such as deforestation, natural disturbances, or changes in plants’ phenological stages [29]. Within the defined watershed 90 reference points (30 points for each vegetation class) from a Google Earth image (CNES/Airbus) of August 2019 [23] were taken and compared with respective NDVI pixel values of a Landsat image of the same date in order to define the NDVI classes of the study site. NDVI values < 0.22 were grouped as non-vegetative areas, NDVI values of 0.22–0.49 defined the class of sparse vegetation (grassland, shrubs and bushes), and areas with NDVI values > 0.49 included dense vegetation such as cropland and trees. NDVI outcomes of unirrigated land were compared to data on local precipitation. Changes in NDVI classes were calculated by determining the total area (number of pixels) of each class for all Landsat images and comparing the area between years.

#### Determination of land use changes

**Fig 5:**
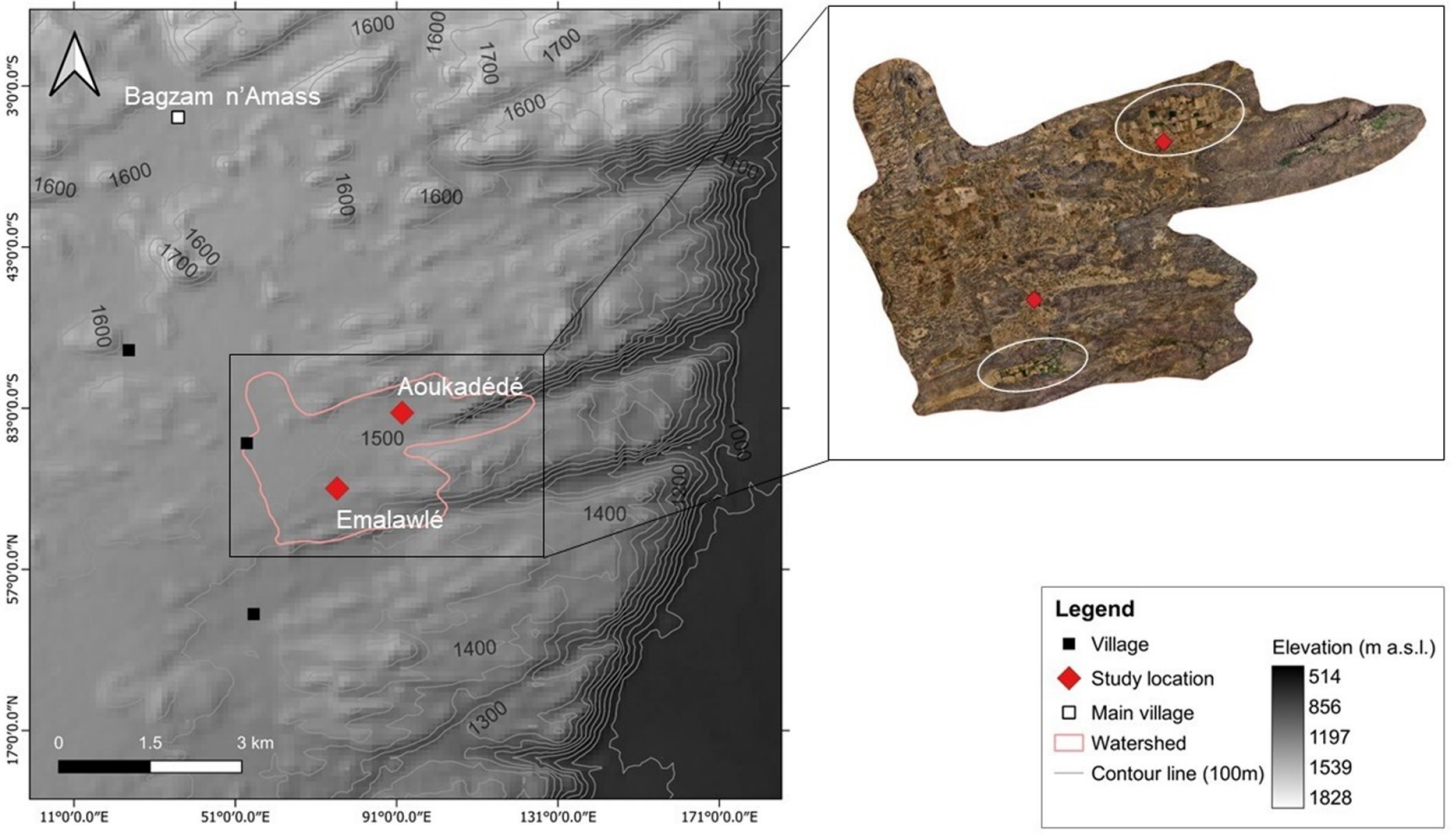
Elevation information and orthomosaic of study area. DEM (2012, [22]) and orthomosaic of drone images taken in August 2022 for the selected 820 ha watershed with marked location (white circles) of the old oasis fields of Emalawlé and Aoukadédé on Mont Bagzam, Niger.

To quantify time courses of changes in land use cover in the oases, georeferenced aerial photographs from the 1950s, Corona satellite images from the 1960s, Google Earth images from 2003, 2016, and 2019 as well as drone images from 2022 were classified manually for the selected watershed [30] (Fig 5) since supervised classification approaches were ineffective in comparing the grayscale and true color images of different resolutions. Furthermore, an identification of field boundaries and recently cleared land for cropping was impractical due to their high similarity to the surrounding land cover and the collection of Ground Truth Points (GTPs) was only possible in the years 2021 and 2022.

Together with the collection of 40 GTPs distributed across the area and the recording of all field boundaries in April/May 2021 using a handheld GPS [20], land use classes were defined as follows: field boundaries, wells, agricultural land (cultivated), uncultivated agricultural land (including fallow and abandoned land), built-up area, and unpaved roads. After manual drawing of points and polygons for the different land use types in each image, temporal changes in land use were calculated by comparing the areas between years. The results were verified with NDVI calculations of the respective time intervals.

#### Agricultural production patterns

To investigate farm-based causes for land use changes on Mont Bagzam as a result of market demand-driven production of onion, garlic, and potato, all gardens of the study region were visited, and a total of 35 household surveys were conducted (Aoukadédé: 24, Emalawlé: 11). The farming households were asked about property rights, ages of their gardens, agricultural practices including inputs and yields as well as about temporal changes in cropping patterns (S1 File). The answers were analyzed descriptively and matched with the results of the remote sensing analyses. Census data, agricultural statistics, and local production records were digitized whereby to our advantage exports of agricultural produce from Mont Bagzam to Agadez have been recorded since 2020 in the storage facility of Tokadi at the foothills of the plateau.

#### Irrigation water needs for newly established fields

To assess the effects of land use changes on irrigation water needs (IN) for cash crop production, the amount of additional evapotranspiration demands of onion and garlic was calculated for the newly cultivated area from 2003 onwards, since before that date crop production was subsistence-oriented.

To this end, the IN (mm year^-1^) was estimated for crop water supply by a combination of irrigation and rainfall [31]:

$$IN={ET}_{c}-P_{e}$$ (10)

where ET_c_ (mm year^-1^) is the potential crop evapotranspiration and P_e_ (mm year^-1^) the yearly sum of effective precipitation.

The yearly irrigation water needs of the additional irrigated area (SIN, l year^-1^) was calculated after [32] assuming optimal crop growth conditions and no irrigation water losses:

SIN=AreaxIN (11)

where Area (m^2^) refers to the difference in cropping land between 2003 and 2022 and IN (l m^-2^ year^-1^) the annual irrigation needs for onion and garlic fields.

#### Soil properties

Soil samples of 27 onion field gardens with an average size of 0.6 ha in Emalawlé and Aoukadédé representing different classes of cultivation ages were collected in 2021 and 2022 to get a preliminary idea about the soil conditions and to generate a false-time-series by analyzing changes in soil properties over time. Four newly established and still uncultivated fields were sampled, 13 fields of ≤ 1 year of cultivation, three fields of 3 years, one field of 4 years, one field of 7 years of cultivation, three fields cultivated for 10 years, and one field each cultivated for 11 years, 12 years, and over 40 years. All crops in these field gardens were amended with a combination of mineral fertilizer (NPK 15:15:15 and Urea 46%) plus an unquantified amount of sheep and goat manure, cultivated, and harvested manually. Soil collection was performed by extracting five samples (0–10 cm) diagonally in each field before pooling. All samples were analyzed for soil pH, soil organic carbon (SOC), carbon/nitrogen (C/N) ratio, electrical conductivity (EC), exchangeable and extractable phosphorus (P) and potassium (K), and extractable calcium (Ca), magnesium (Mg), sodium (Na), iron (Fe), and aluminum (Al) as described elsewhere [33].

Prior to analyses all soil samples were air-dried, sieved to < 2 mm particle size, and visible plant roots were removed. Soil pH was determined at a soil/water ratio of 1/2.5 by a glass electrode (ProLab 1000, SI Analytics GmbH, Mainz, Germany). EC was recorded using a digital conductivity meter (3430, GHM Messtechnik GmbH, Regenstauf, Germany) at a soil/water ratio of 1/2.5. Soil total C and N were measured by a thermal conductivity detector (Vario MAX CHN Analyser, Elementar Analysensysteme GmbH, Hanau, Germany). SOC was determined by deducting the soil inorganic carbon content (CaCO_3_ and MgCO_3_), measured by a calcimeter, from the total carbon content. Extractable nutrient contents in the soil including P, K, Ca, Mg, Na, Fe, and Al were measured with an Inductively Coupled Plasma–Optical Emission Spectrometry (ICP–OES), after an *Aqua Regia* digestion. Plant available (exchangeable) P and K were determined with the ICP–OES after a calcium-acetate-lactate (CAL) extraction [34].

Due to an overall small sample size and varying sample numbers per class of cultivation age, simple linear correlation analyses and graphs were performed using the statistics software RStudio (vers. 9.1.191) to determine the relationship between different durations of cultivation and key soil parameters, defined by the Pearson correlation coefficient at p < 0.05. Soil salinity was expected to increase with duration of field cultivation given a negative water balance between evapotranspiration and precipitation at regular irrigation. SOC was expected to be low given the only recent onset of soil cultivation, sparse natural vegetation, and high turnover rates of organic carbon due to the prevailing climatic conditions [35].

#### Ethical authorization

The study was authorized by the Ministre de l’Enseignement Superieur et de la Recherche, Niamey, Niger under Permit N°00206 of 28/02/2022 while the data collection procedure and subsequent use of the data was approved by the Central Ethics Committee of the University of Kassel, Germany.

## Results

### Land use changes

Since the construction of an unpaved road in 2015 connecting the villages of Mont Bagzam to the major regional marketplace of Agadez, agricultural production expanded and several new gardens cultivated with cash crops evolved, not only in the watershed between Aoukadédé and Emalawlé, but on the entire Mont Bagzam. A multitude of new wells were established, operated with diesel water pumps, for the irrigation of new, comparatively large-scale fields with cash crops between the old oasis centers.

**Fig 6:**
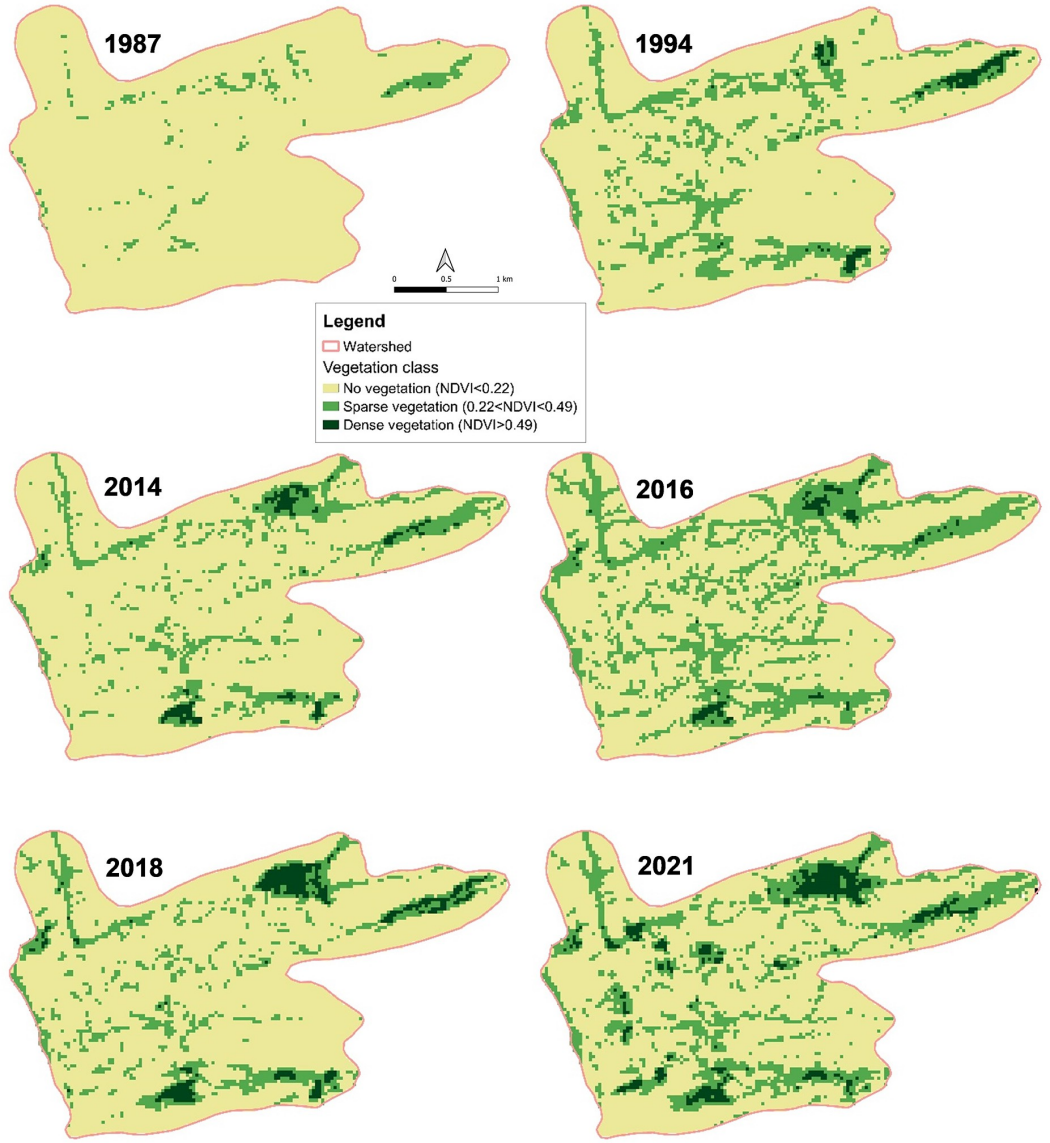
Normalized Difference Vegetation Index (NDVI) maps for a 44 years’ time span. NDVI of Landsat 5 and 8 images from 1987–2021 of the selected watershed on Mont Bagzam, Niger.

The NDVI analyses of Landsat images of the selected watershed of Emalawlé and Aoukadédé showed an increase in the area of dense vegetation (cropland and trees) from 0.3 ha in 1987 and 13.2 ha in 1994 to 15.3 ha in 2014 and 12.2 ha in 2016, to 33.5 ha in 2018, and 42.8 ha in 2021 (Fig 6). The area covered by sparse vegetation (shrubs and grassland) increased from 31 ha in 1987 to 193 ha in 2021. While size and location of dense vegetation only slightly varied between 1994 and 2018, since 2018 there has been a clear rise of dense vegetation in the area between and west of the two oasis centers.

**Fig 7:**
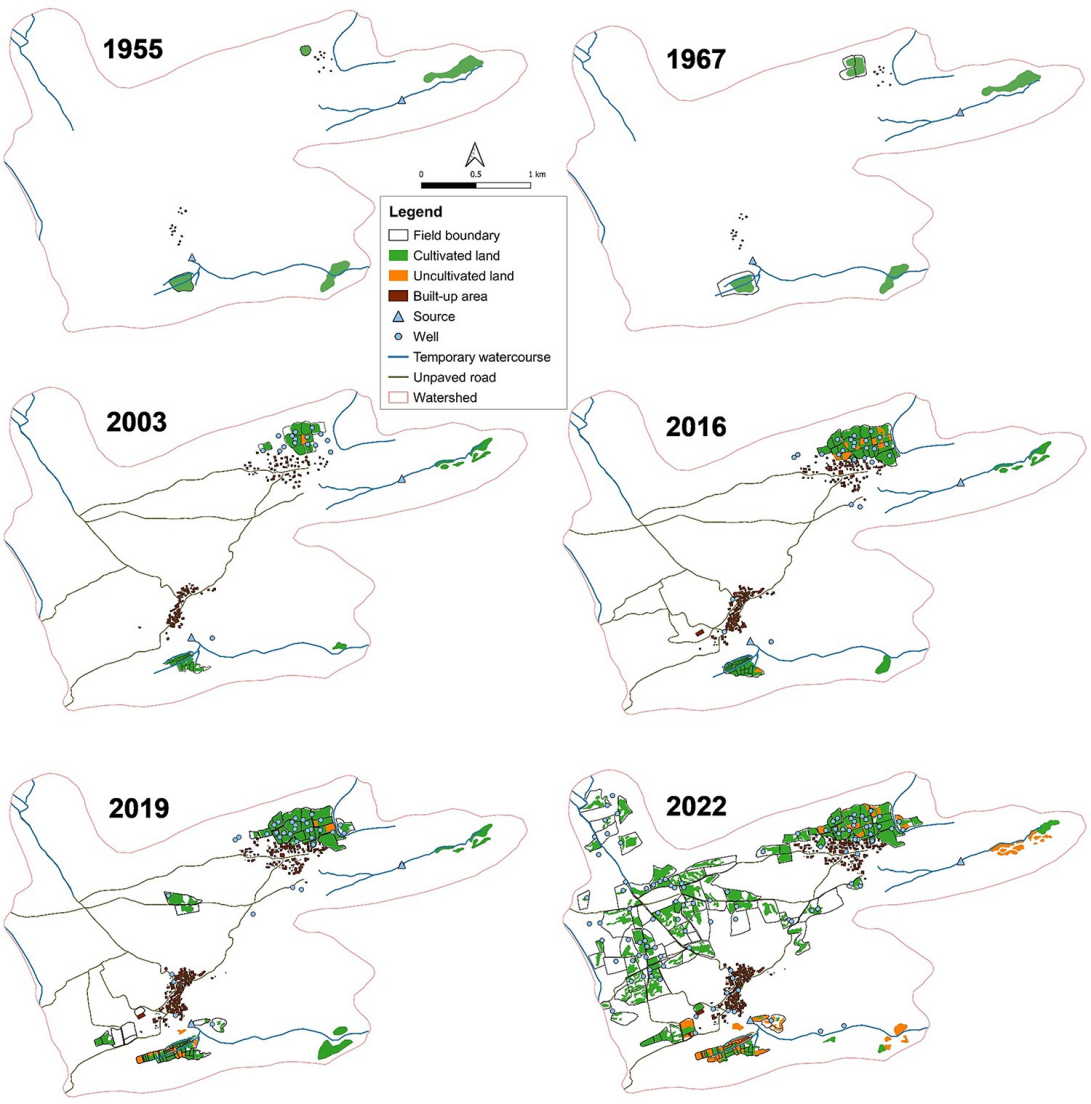
Land use maps for a 67 years’ time span. Land use changes of Emalawlé and Aoukadédé, Mont Bagzam, Niger between 1955 and 2022 based on manual image classifications.

For the time span of 1955 until 2003 (almost 50 years) the results of manual land use classifications indicated an increase in cultivated land area of only 20%, while during the past eight years land under cultivation has tripled. Prior to 2000 the inhabitants lived in small tent type structures and clay houses, whereas from 2003 onwards increasingly cemented structures using stones and loam were identified. Since 2014 the watershed of Emalawlé and Aoukadédé underwent massive changes in agricultural land and groundwater use with sprawling new fields in formerly lawa rubble-covered areas (Fig 7).

Field sizes in the old oasis of Emalawlé averaged 0.25 ha, whereas newly established fields since 2016, surrounded by protective fences, have average sizes of 1.7 ha. Most of the newly established fields belong to the population of Emalawlé who managed to clear the newly acquired land from stones and bushes with the help of cheap hired labor from Houssa lands in southern Niger or Nigeria (Table 4).

**Table 4:** Calculated differences in land use between 2003 and 2022. Differences in land use of Emalawlé and Aoukadédé, Mont Bagzam, Niger between 2003 and 2022 based on manual image classifications of built-up area and agricultural land.

	Area (ha) in 2003	Area (ha) in 2022	Δ Area (ha)
Buildings	4	9	+ 5
Field boundaries	12	157	+ 145
Cultivated land	13	68	+55
Uncultivated land	< 1	15	+14

Traditional palm groves and fruit trees are only cultivated in the old oasis centers, whereby the fruits of 470 palm trees counted in 2022 serve no longer as an income source since their manual fertilization and harvest is too costly compared to the procurement of higher quality imported dates. Typically, every new field has an individual well, up to 20 m deep and operated by a motor pump. From 2003 to 2022 the total number of operational wells in the watershed has increased from 16 to 108.

**Fig 8:**
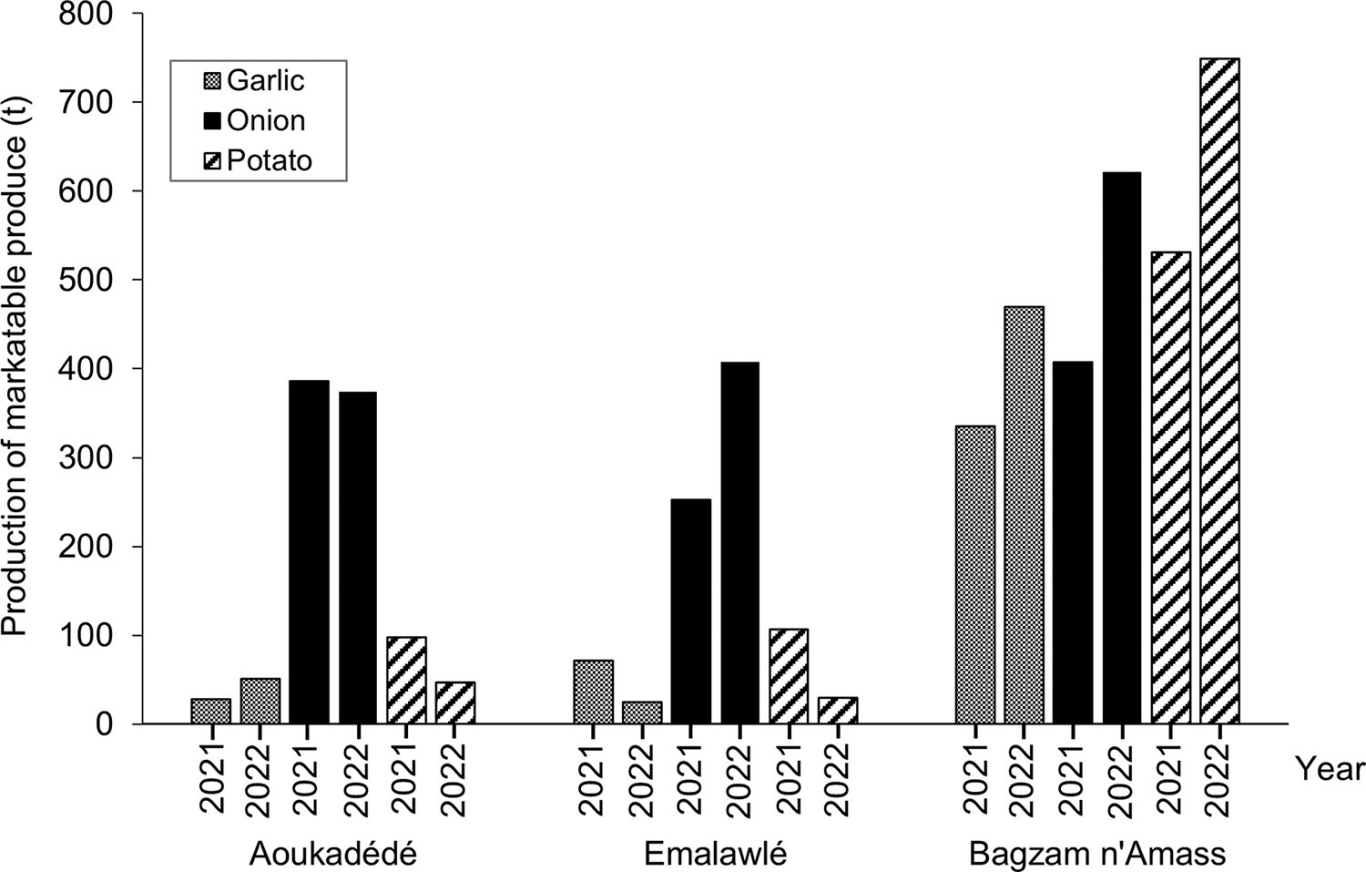
Cash crop production amounts. Production of marketable produce (onion, garlic, potato) of the villages Aoukadédé, Bagzam n’Amass and Emalawlé on Mont Bagzam, Niger from May 2020 –May 2022 (S1 Table).

The records of agricultural produce in the storage facility at Tokadi showed that garlic, onion, and potato are the only exported products from Mont Bagzam (Fig 8). In the selected two oases Emalawlé and Aoukadédé especially onions are produced in large quantities. From 2020–2022 the exported onion volume of Emalawlé rose by 60% while garlic and potato volumes slightly decreased. Farmer-reported reasons are yield losses of garlic due to diseases and a decreasing availability of irrigation water. While overall crop production in Aoukadédé slightly decreased during 2020–2022, the production volume of cash crops in Bagzam n’Amass has increased.

### Evapotranspiration and changing irrigation water need

**Fig 9:**
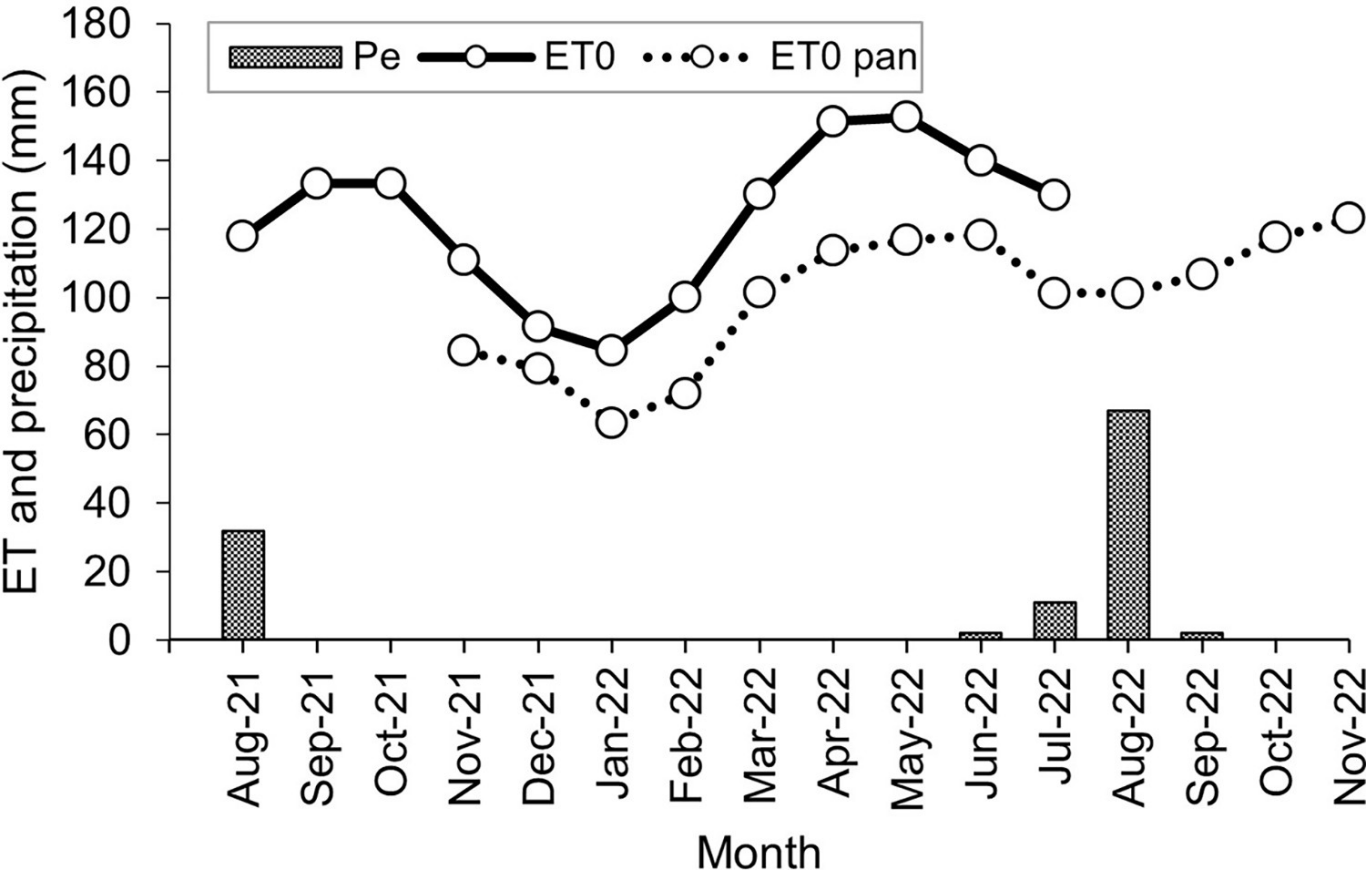
Evapotranspiration. Effective precipitation (P_e_), potential reference evapotranspiration (ET_0_), and actual pan reference evapotranspiration (ET_0 pan_) from August 2021 –November 2022 in Emalawlé, Mont Bagzam, Niger.

Annual reference evapotranspiration (ET_0_) calculated by climatic data from August 2021 –August 2022 amounted to 1,476 mm year^-1^. Monthly ET_0_ was highest in May with 153 mm and lowest in January with 85 mm. Actual pan evapotranspiration (ET_pan_) was measured from December 2021 –December 2022 and amounted to 2,699 mm year^-1^. The derived actual ET_0 pan_ was 1,215 mm year^-1^, thus 18% lower than the potential ET_0_ derived from climatic data (Fig 9).

ET_c_ of onion and garlic was calculated as 680 mm and 630 mm year^-1^ respectively. Total yearly IN of onion and garlic therefore amounted to 1,265 l m^-^^2^. With the additional cultivated land area of 55 ha irrigation annual water needs rose by 695.8 million l.

### Soil quality

**Fig 10:**
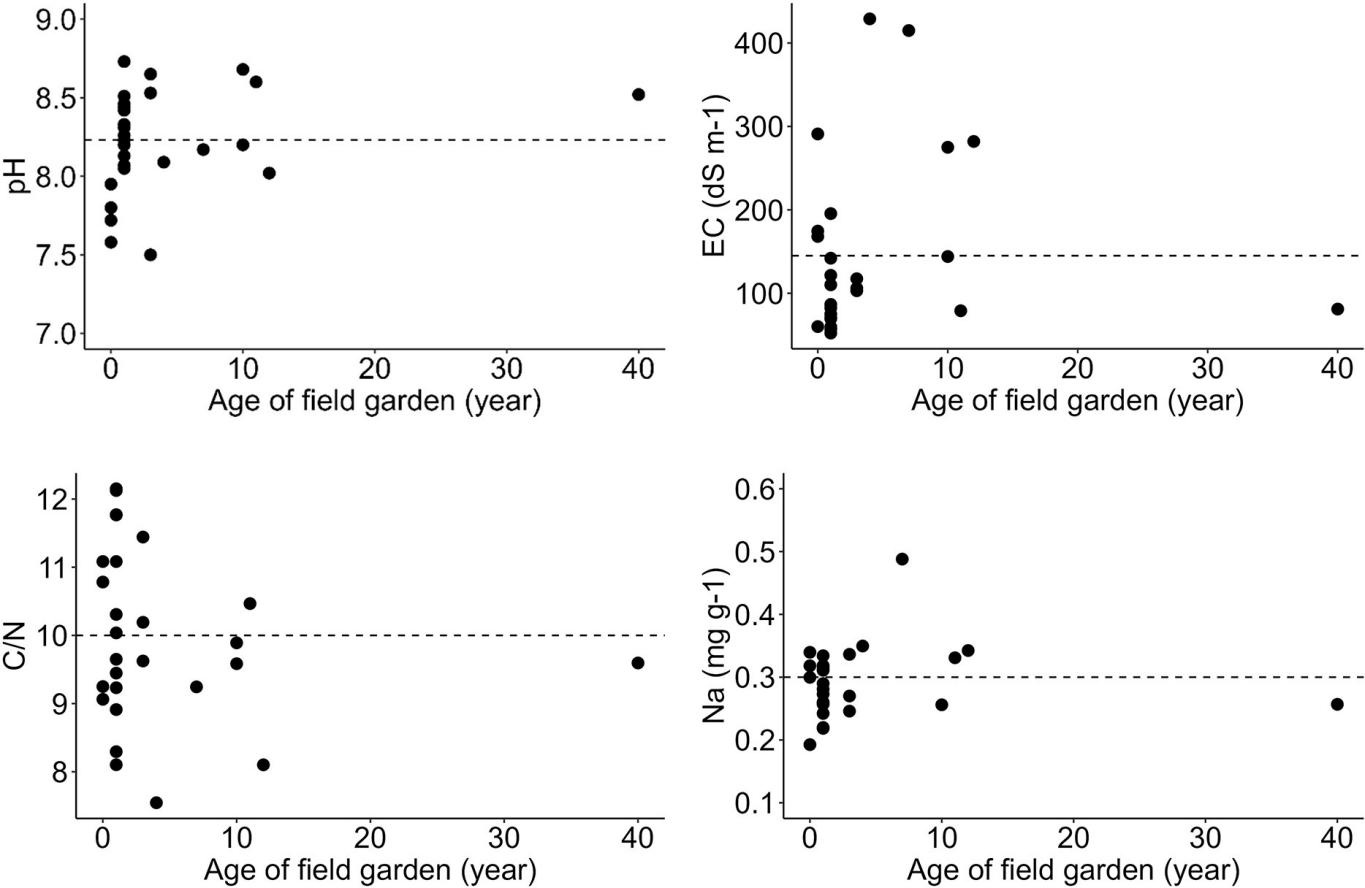
Key soil property values in relation to ages of field gardens. Relationship between cultivation period (0 years = uncultivated control group) and soil pH, EC (dS m^-1^), C/N ratio, and extractable Na concentration (mg g^-1^) (with means indicated as dotted lines) in fields of Emalawlé and Aoukadédé, Mont Bagzam, Niger (S2 Table).

Our soil sample analyses of onion and garlic fields show an average pH of 8.23, which confirms the alkalinity of soils on Mont Bagzam. Correlation analyses revealed that differences in pH, EC, C/N ratios, and Na levels cannot be clearly associated with the duration of cultivation (p > 0.05 in all cases). However, there was a tendency of increasing pH and Na over time (Fig 10). Especially extractable Na concentrations of newly established fields (1–10 years) seem to increase with cultivation time, even if the field of > 40 years had surprisingly low Na concentrations.

C/N ratios slightly declined with cultivation time of newly established fields whereby SOM and SOC contents were generally low (mean SOM = 3.32%, mean SOC = 0.64%). Exchangeable P slightly increased over time, whereas exchangeable K declined (Table 5). All soil samples showed high levels of Al (35.5 mg g^-1^ soil on average) and Fe (28.05 mg g^-1^ soil in average) reflecting long periods of basalt weathering.

**Table 5:** Overview of soil chemical properties. Mean values for soil chemical properties (0–10 cm) with +/- one standard deviation in parentheses and grouped after duration of cultivation (0 years = uncultivated control group) of selected onion and garlic fields (n = 27) in the oases of Emalawlé and Aoukadédé, Mont Bagzam, Niger (2021–2022).

Ages of garden fields (years)	Number of samples (n)	SOM (%)	SOC (%)	Ca (mg g^-1^)	Mg (mg g^-1^)	P* (mg g^-1^)	K* (mg g^-1^)
0	4	3.69 (1.25)	0.56 (0.24)	5.26 (0.63)	5.60 (0.89)	0.16 (0.05)	0.67 (0.39)
1	13	3.18 (0.85)	0.42 (0.10)	4.76 (0.89)	4.71 (1.10)	0.16 (0.09)	0.46 (0.11)
3	3	3.04 (0.06)	0.65 (0.11)	6.65 (2.18)	4.33 (0.77)	0.26 (0.07)	0.41 (0.07)
4	1	3.29	0.66	4.89	3.12	0.18	0.78
7	1	3.21	0.65	5.89	3.51	0.32	0.57
10	2	3.34 (0.84)	0.72 (0.38)	5.20 (0.21)	3.57 (0.12)	0.21 (0.08)	0.35 (0.11)
11	1	3.43	0.56	5.13	4.51	0.05	0.33
12	1	2.87	0.49	4.59	3.11	0.29	0.47
40	1	3.23	0.93	4.82	3.29	0.19	0.4

* Plant available (exchangeable) nutrients as determined by calcium-acetate-lactate (CAL) extraction.

## Discussion

The results of our remote sensing analyses combined with ground truthing provide evidence of massive changes in the 820 ha watershed of Emalawlé and Aoukadédé within the past seven years. Prior to road construction in 2015 agricultural activities were determined by subsistence needs of the local population. Therefore, crop production was dominated by cereals and vegetables cultivated on small terraces. Dates, millet, and surplus animals were traded against salt and other necessities by camel and donkey [13,15]. After the establishment of the road connection to the trading hub of Agadez, the area used for agriculture has quintupled.

These human-induced changes were reflected in our time series analyses of NDVI values and LUC data obtained from manual classification of agricultural fields. However, NDVI analyses also showed that the distribution and quality of sparse vegetation, apart from human and grazing activities, heavily depend on the amount and distribution of rainfall in the year of analysis and the precedent one. These dependencies are in line with other studies applying NDVI for identifying LUC [36,37]. The high rainfall in 2015 and 2020 are reflected in area increases of sparse vegetation in 2016 and 2021. Nevertheless, the high density vegetation largely developed in an area of the watershed that has remained uncultivated prior to 2014.

Data on exported agricultural produce from Mont Bagzam reflects the reported changes in land use whereby a doubling of agricultural land in Emalawlé between 2020 and 2022 led to an increase in marketable onion by 60% in the respective time interval. The focus on onion production coincides with the rising national net export numbers for onion to meet market demands of neighboring countries, especially Ghana, Ivory Coast, and Benin [38,39]. Even though research is lacking on the magnitude and environmental effects of accompanied LUCs in Niger, studies in other Saharan countries confirm growing areas of irrigated agriculture for cash crop production leading to water shortages and salinization problems [40,41].

Rather than climate change, stated as a major threat for agricultural systems in other arid and semi-arid regions [42,43], the rising overuse of common pool resources by farmers on Mont Bagzam, reflecting what is known as the “tragedy of the commons” [44], questions future land productivity and the security of livelihoods. The original concept of destruction of the commons has been further developed [45] and used in various studies on the stability of small-scale societies, when rapid regime shifts as a consequence of telecoupled changes in resource demands occur [46–48]. A major challenge for the region on Mont Bagzam, whose inhabitants’ survival has depended on sustainably and commonly managed resources since millenia, became the unregulated appropriation of private land and groundwater harvesting in former common rangelands after the area’s inclusion in the modern market economy. Increasing abandonment and fallow periods of terraces in the old oasis centers reflect water shortages due to indiscriminate agricultural expansion and intensification of new uphill locations at the agricultural frontiers. Expanded cultivation at the topographically higher location of Bagzam n’Amass hampers water availability of Emalawlé and Aoukadédé. At the same time, new fields of Emalawlé were established closer to the main water inflow and further impact water availability in Aoukadédé and old fields of Emalawlé, which seems also reflected in Aoukadédé’s slightly declining agricultural production volumes in 2022 compared with 2021.

The focus on growing cash crops on an area that grew by 55 ha in the last two decades requires annual application of additional 696 million l irrigation watercompared with small-scale and low-input subsistence farming before 2014 on 13 ha. The ongoing expansion of land and the related construction of wells have already led to a reported falling of groundwater levels across the plateau of Mont Bagzam and drying up of village wells. Given a yearly ratio of P_e_/ET_0_ = 1/10 and the reliance on rainwater for irrigation, sustainable intensive agriculture under surface irrigation will require reliance on a large impluvium area and improved retention of runoff water from the temporary water streams (“Wadi” or “Kori”) that drain Mont Bagzam’s floodwaters to the foothills. The agricultural exploitation of large areas cleared from bushes and stones results in increasing risks of runoff leading to enhanced soil erosion and flooding events as demonstrated by other studies on mismanagement in dryland farming systems [49,50].

Soil sample analyses of Aoukadédé and newly established fields of Emalawlé revealed slightly increasing pH levels and trends for rising Na concentrations in the topsoil with cultivation time. The observed imbalance between rainfall, evapotranspiration, and water inputs as well as the unsuitability of monocropping cash crops under these conditions may be reflected in rising pH levels, EC, and Na concentrations in the future [51]. Our survey data reveal that fields of the traditional oasis centers are mainly fertilized with manure and only small amounts of mineral fertilizers to wheat and onion, while most new fields receive comparatively lower amounts of manure, which is reflected by low SOC contents and faster decomposition rates.

To ensure future livelihoods on Mont Bagzam, maintenance of agricultural production on increasing fields will require introduction and widespread use of water harvesting approaches such as deviation of runoff water and interception in cisterns or “false wells” and the application of drip irrigation and mulching. The effectivity of such water and land care approaches has been successfully demonstrated in the Middle East [52,53], but also more recently in Sub-Saharan countries [54]. In this context the use of agroforestry systems with intercropping of trees and cash crops on the newly established fields may increase agricultural productivity while reducing risks of soil salinization and erosion [55].

Onions produced on Mont Bagzam are a prime example of how telecoupling of production and consumption driven by booming urban markets of the sprawling West African coastal cities across distances > 2500 km under very difficult infrastructural conditions offers new livelihood opportunities [39]. In a wider context it demonstrates that even the most rural regions of Africa, such as the oases on Mont Bagzam at the southern fringe of the great Ténéré Desert, no longer exist in isolation, but have become part of regional and global food networks [39,56]. These new opportunities, however, are coupled with major risks for the resilience of the social-ecological systems and the sustainability of ecosystem services [7].

Even though the identified changes in agricultural production and implications for water use and soil fertility within this study applying a mixed-methods approach seem consistent, further studies are needed. Detailed climatic data have only been recorded for one year and soil parameters and changes using a false-time-series approach were determined on a limited number of locations with IN being calculated by using a simplified approach. To further assess implications of land use transformation for these marginal agroecosystems over time, investigations should also be done on available groundwater resources. Water use efficiency needs to be calculated field- and crop-based and field tests are needed to monitor effects of alternative cropping systems using drip irrigations and agroforestry gardening for cash crop production.

## Conclusions

Transformation of oasis systems on Mont Bagzam in Niger is triggered by rapid growth of the local population and new marketing opportunities in remote urban centers for high quality, easily storable, and bulk-transportable cash crops thriving under desert conditions. While in the short-term, rural populations on Mont Bagzam take great advantage of this new income opportunities, our data indicate that in the longer-term over-exploitation of rain-dependent water resources and declining soil fertility threatens sustainable agricultural land use and social coherence. The far-reaching telecoupling effects of global food supply networks on rural marginal areas are often neglected although they may severely endanger the preservation of biodiversity, traditional knowledge, and ultimately cultural identity. Therefore, more integrated trans-boundary approaches of land use and related policies are required that foster the economically profitable and sustainable use of marginal areas with highly fragile ecologies that undergo rapid regime shifts as a consequence of global, urbanization-driven rural-urban transformation.

## Supporting information

S1 Table Registered amounts of marketable produce from Mont Bagzam, Niger. Quantities of onion, garlic and potato registered at the storage facility of Tokadi from May 2020 to May 2022.
(XLSX)

S2 Table Soil chemical properties of field gardens on Mont Bagzam, Niger. Soil chemical properties (0–10 cm) of selected onion and garlic fields (n = 27) in the oases of Emalawlé and Aoukadédé taken in 2021 and 2022.
(XLSX)

S1 File Survey questions for Emalawlé and Aoukadédé, Mont Bagzam, Niger. (PDF)
