# Supplementary material for: Telecoupled urban demand from West African cities causes social-ecological land use transformation in Saharan oases
Source: PLoS One. 2023 Sep 8;18(9):e0289694. doi: 10.1371/journal.pone.0289694 (PMC10490980; doi:10.1371/journal.pone.0289694)
Supplement: S1 File — (PDF) [file pone.0289694.s003.pdf]

Survey questions for Emalawlé and Aoukadédé, Mont Bagzam, Niger

1. Household (socio-economic)
2. Agricultural workforce
3. Crop/livestock land use
4. Crop production
5. Crop marketing
6. Migration

Household ID:

1. Household (socio-economic)

1.1 Person Record

Number	Age	Gender	Marital status	Education	Occupation	Residency	Participation in farm work (Y/N)	Hours/Week

1.2 Household Record

Q: What is the total household income?

Q: What is the share of income from agriculture (marketing) CROP?

Q: What is the share of income from agriculture (marketing) LIVESTOCK?

Q: What is the share of income from off-farm activities?

Q: What are the off-farm activities and since when are they practiced?

2. Agricultural Workforce

Q: How much time do you spend for the care of your crops (h/day)?

Q: How much time do you spend for the care of your livestock (h/day)?

Q: Do you use hired labor (Y/N)?

If YES:

Persons	From where?	Payment (per day)

Q: Do you want to continue farming in the future (Y/N)?

If YES, why?

If NO, why not?

Q: Do you want your children to become a farmer (Y/N)?

If YES, why?

If NO, why not?

3. Crop/Livestock land use

Q: How much land do you own?

Q: Where is your land (show on the map/No. of plots)?

Q: How much land do you use for CROP cultivation?

Q: Has your cropping land increased/decreased in the past 3/5/10/20 years?

Time span	Change of crop land size (+/0/-)	What is the reason for change in crop land size? (Environmental conditions/Buildings for own use/Livestock farming/ Higher sales/Other)
3		
5		
10		
20		

Q: How much land do you use for grazing?

Q: How much of your grazing land do you use for fodder production?

Q: How much FALLOW LAND do you have?

Q: Has this uncultivated area size changed over time?

Time span	Change (+/0/-)	Reason
3		
5		
10		
20		

Q: Do you rent additional plots (Y/N)?

If YES:

How much?	From whom?	Reason
	Neighbor	
	Communal Land	
	Relatives	
	Other	

Q: Do you rent out plots (Y/N)?

If YES:

How much?	To whom?	Reason
	Neighbor	
	Communal Land	
	Relatives	
	Other	

4. Crop production

4.1 Variety

Q: Which crops do you cultivate? How did they change over time?

Species	Plots	Yield	Season (crop rotation)	% sold	% own consumption	Change in the past 1/3/5/10 years (increase/decrease/ no change)	Reason for cultivation (HH consumption/Marketing/Fodder/Storage ability)

Q: Which crops are most important for you?

Crop species
1.
2.
3.

Q: Where do you get seeds from? / Do you (re-)grow your own seeds?

Crop	Origin

Q: What are the main problems with crop cultivation?

Time	Problem 1	Problem 2	Problem 3
Past			
Today			
Future			

4.2 Intensity/Input

Q: Do you use manure/compost on your fields (Y/N)?

If YES, for which crops?

Quantity per unit area / plot	
From which animals?	
% own production	
% bought	

Q: Do you use mineral fertilizer (Y/N)?

If YES, for which crops and on which plots?

What kind of fertilizer?	
Quantity? (total per unit area)	
When do you apply it?	
Price per unit	

Q: Do you irrigate your fields?

If YES:

What is your source of water?	
How many days per week do you irrigate your fields?	
Which technique? (drip/flood/canals)	
Do you have to pay for the water?	
Has your source of water changed within the last 10 years (Y/N)?	Yes No
If YES: From to?	

5. Crop marketing

Q: Which crops and processed products do you sell?

Crop	Product	Where do you sell?	Distance from the farm?	To whom? (locals, cooperative, wholesaler)	Unit of sale	Price per unit	Selling season	Units sold per year/season

Q: Do you sell more or less than 1, 3, 5, 10 years ago?

Crop/Product	1 year	3 years	5 years	10 years	Reason

Q: Has your marketing changed over time (Y/N)?

If YES, in which way?

Q: Do you have problems with marketing (Y/N)?

If YES, which difficulties do you have? (market access, seasonality, work force)

8. Migration

Q: Do you or your relatives migrate seasonally to other cities/states for employment?

If YES:

Who?	Destination	Time frame	Reason

Q: Did you or your relatives migrate seasonally to other cities/states for employment in the past (before Gaddafi's death)?

If YES:

Who?	Destination	Time frame	Reason

Q: Do you or your relatives practice caravan trade for salt?

If YES:

Who?	Destination	Times per year	Reason

Thank you for your participation!
